# Phylogenetic Reconstruction of Aging Rates in the Primate Lineage

**DOI:** 10.1093/geroni/igaf122.2302

**Published:** 2025-12-31

**Authors:** Eugene Melamud, Wendy Newton, Joseph Kemnitz

**Affiliations:** Calico Labs, Calico Labs, California, United States; CleMetric, Madison, Wisconsin, United States; University of Wisconsin–Madison, Madison, Wisconsin, United States

## Abstract

Median lifespans of primates show nearly 10-fold variation, ranging from ∼8 years in marmosets to ∼80 years in humans. The molecular mechanisms that govern this variation and how they evolved remain poorly understood. In this study, we implemented a novel phylogenetic Gompertzian survival framework to leverage the evolutionary history of primates in order to estimate parameters of aging for 38 captive primate species. We find that baseline hazards (at the time of sexual maturity) and adult aging rates display significant variation, and aging rates are more evolutionarily conserved than baseline hazards (Pagel lambda=0.96 vs lambda=0.34, respectively). Furthermore, we find that aging rates and baseline hazards do not show a pattern of phylogenetic covariation, suggesting differential evolutionary pressures may act on these traits. Aging rates were strongly correlated with body weights, with a notable exception within the Ape family, where aging rates remained approximately unchanged despite large differences in body weight. Based on the reconstruction of ancestral aging rates, we estimate that the ancestor of Apes was likely aging at a similar rate to modern humans.

